# Unveiling insights from the Joint FAO/WHO Expert Committee on Food Additives (JECFA) portal

**DOI:** 10.1038/s41597-024-04294-w

**Published:** 2024-12-28

**Authors:** Honoria Ocagli, Corrado Lanera, Marco Franzoi, Chiara Monachesi, Rebecca Zgheib, Simone Belluco, Mauro Dacasto, Dario Gregori, Ileana Baldi

**Affiliations:** 1 Unit of Biostatistics, Epidemiology, and Public Health, Department of Cardiac Thoracic Vascular Sciences and Public Health, University of Padova, via Loredan 18, Padova, 35131 Italy; 2 Laboratory of Food Chain Safety and Quality, Istituto Zooprofilattico Sperimentale Delle Venezie, Viale Fiume 78, 36100 Vicenza, VI Italy; 3 Department of Comparative Biomedicine and Food Science, University of Padua, Viale dell’Università 16, Legnaro, 35020 Padua Italy

**Keywords:** Chemical biology, Research data

## Abstract

This study presents a method for automating the retrieval of key identifies and links to toxicological data from the Joint FAO/WHO Expert Committee on Food Additives (JECFA) database using web scraping techniques. Although the method primarily serves as an automated indexing tool, facilitating organization and access to relevant reports, monographs, and specifications, it significantly enhances the efficiency of navigating the extensive JECFA database. Researchers can then perform more targeted and efficient searches, although additional manual steps are required to extract and structure the detailed toxicological data. We developed R programming scripts to extract key information, such as chemical names, identifiers, and evaluation reports, from JECFA web pages. The resulting data set comprises 6552 records as of May 2024. We validated the dataset through a systematic comparison with manually collected data, ensuring its reliability. Instructions and code for accessing and processing the dataset, facilitating its reuse in research are provided. The code and dataset are openly available, enabling researchers to efficiently access and analyze toxicological data from the JECFA database.

## Background & Summary

Chemicals pose significant environmental threats to human health, underscoring the need for a comprehensive understanding of hazards and exposure data. Such data, often referred to as the chemical exposome, are essential to assess health risks and prevent disease^1^.

Toxicology has evolved from an empirical science focused on observing the effects of chemical exposure to a data-rich field^2,3^. A growing body of electronic databases (DBs) and repositories assists in the storage, organization, and access of data on the toxicological effects of chemicals, including data from toxicology studies, risk assessments, and regulatory decisions. A recent literature review identified 362 toxicological DBs providing a broad range of information such as physicochemical properties, experimental studies results, reference/limit values (e.g., cancer slope factor), prediction data, prediction models, dose response models or other toxicity information^4^.

Risk assessment is central to regulatory decision making in toxicology. Public databases like the European Food Safety Authority’s (EFSA) OpenFoodTox^5^ and the U.S. Environmental Protection Agency’s (EPA) IRIS-EPA^6^ consolidate toxicity data for regulatory use, providing summaries of hazard identification and characterization data for various assessments.

Established by the FAO (Food and Agriculture Organization of the United Nations) and the WHO (World Health Organization), the Joint FAO/WHO Expert Committee on Food Additives (JECFA) stands as a prominent global scientific entity, widely acknowledged for its pivotal role in evaluating the safety and functionality of food additives. It operates at the convergence of scientific expertise and global health policy^7^.

As an independent scientific expert committee, JECFA conducts risk assessments and offers guidance to the member countries of both organizations and the Codex Alimentarius Commission (CAC). Their searchable database encompasses summaries of JECFA’s evaluations, covering flavors, food additives, contaminants, toxicants, and veterinary drugs. Each summary includes basic chemical information, identifiers, acceptable daily intake (ADI) and/or tolerable daily intake (TDI), links to supporting documents, and a history of JECFA evaluations.

WHO and FAO are responsible for the preparation of toxicological monographs in the Food Additives Series (FAS) and reports in the Technical Report Series (TRS) related to chemicals under investigation. These documents are publicly accessible on their official website^8^, to which the JECFA database is linked.

Unlike OpenFoodTox and IRIS-EPA, the JECFA database cannot be downloaded. Furthermore, JECFA does not offer an accessible application programming interface (API) to query its database, resulting in a manual and time-consuming process for downloading documentation. This becomes a significant barrier for studying any chemical reported in the JECFA portal.

In addition to the EFSA and EPA databases, other significant resources for structured toxicity data include the OECD eChemPortal (https://www.echemportal.org/echemportal/), which provides access to chemical safety data and regulatory information from participating databases worldwide, and the OECD Toolbox, a platform designed to support the grouping of chemicals into categories to assess their potential health hazards. The EPA’s CompTox Chemicals Dashboard (https://www.epa.gov/comptox-tools/comptox-chemicals-dashboard) also deserves mention, as it aggregates data from multiple sources, including the ToxValDB and ToxRefDB databases, offering extensive information on chemical toxicity. Additionally, resources from organizations such as the FDA and NICEATM (National Toxicology Program Interagency Center for the Evaluation of Alternative Toxicological Methods) (https://ntp.niehs.nih.gov/whatwestudy/niceatm) also provide valuable structured toxicity data that serve various regulatory purposes beyond merely indexing information. These platforms offer comprehensive data that can be directly applied to hazard assessment and regulatory decision-making.

Accessing the information linked to all JECFA assessments is valuable for several reasons, including providing detailed information on hazard identification, hazard characterization, and safety evaluations. These documents provide information on the scientific methodologies and criteria used by JECFA, supporting a better understanding of safety assessments. Moreover, obtaining the link to and downloading the PDFs not only supports research, regulatory compliance, and global harmonization in food safety standards, but also facilitates the application of text mining analyses^2^.

Automated tools can be used to extract relevant information from large databases, such as JECFA, reducing the time and effort required for manual access and data analysis. One prevalent method of gathering digital data is the practice of Web scraping^9,10^. The massive amount of data available on the Web means that effective data collection and processing cannot be performed manually by individual researchers or even large research teams. Web scraping, an alternative to manual data collection, involves using computer programs to automate the extraction and organization of data from the Web for further data analysis and use. Web scraping has also become a valuable tool in epidemiological research and public health planning^11^. Without an API or direct access to the JECFA database, we sought to describe and make available a series of third-party R programming scripts to help automate the acquisition of publicly available JECFA data.

Our study aims to provide researchers with an organized index of chemicals evaluated by JECFA, complete with essential identifiers and direct links to related reports and monographs. While manual querying is still necessary for detailed data extraction, this method streamlines the process of navigating the JECFA database and locating relevant documents^12^.

## Methods

**Fig. 1:**
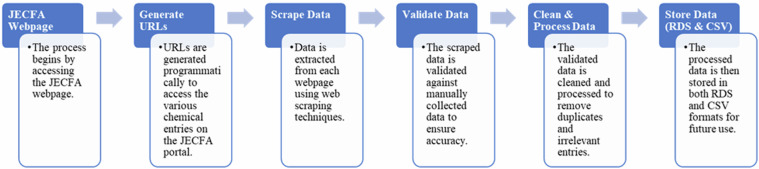
Steps of the project.

In Fig. 1 are reported the steps taken during the data extraction and processing phases.

The JECFA site was explored iteratively by accessing all pages of hosted chemicals (https://apps.who.int/food-additives-contaminants-jecfa-database/) using R software (v. 4.3.2; https://www.R-project.org/) and the packages “httr”^13^ and “rvest”^14^. The data, publicly available on FigShare, reflect the data on the JECFA portal as of May 2024. A GitHub repository contains the source code and the documentation for using the R to efficiently produce the data. The R code has been containerized using Docker^15^, ensuring portability and ease of deployment. The Docker project provides a consistent environment for running the analysis independent of the OS hosting platform and its installed software. Users can execute the code to fetch the most recent data from the JECFA portal, ensuring access to the latest information. Users can execute the code to fetch the most recent data from the JECFA portal, ensuring access to the latest information. The key feature of our pipeline is its efficiency in handling updates. It only processes data that has not yet been processed, meaning it only scrapes and processes new or updated information. This ensures that each run of the pipeline is quick and focused on the most recent data, avoiding unnecessary reprocessing of previously analyzed data. This design allows the pipeline to be executed as frequently as desired, with the assurance that only the “new/updated” data will be incorporated into the database, tables, and final reports. Existing documentation further details this process, ensuring that users can adjust the frequency of updates based on their specific requirements without overloading the system. This setup also ensures reproducibility, providing users with a locked environment that contains the specific versions of the packages used during creation. Using Docker, users can avoid potential issues with package versions and dependencies, ensuring that the code runs seamlessly across different systems. This method facilitates a reliable and user-friendly experience for accessing and analyzing JECFA data.

We have provided a comprehensive README file in the GitHub repository, including general instructions on the project, details on the local and Docker setup, and guidance on executing the pipeline. Additionally, two detailed vignettes vignettes guide users through setting up the environment (both locally and via Docker) and executing the pipeline. They also provide instructions on how to inspect and access the objects generated during the process.

### Web-scraping

Given the absence of APIs for direct data querying, we used URLs as the primary method for data extraction, ensuring consistency and accuracy. The data extracted by this method were then systematically compared with the information available in the original database to ensure completeness and reliability. The alignment between the extracted data and the data file provided was carefully validated to ensure that all relevant fields were accurately represented.

**Fig. 2:**
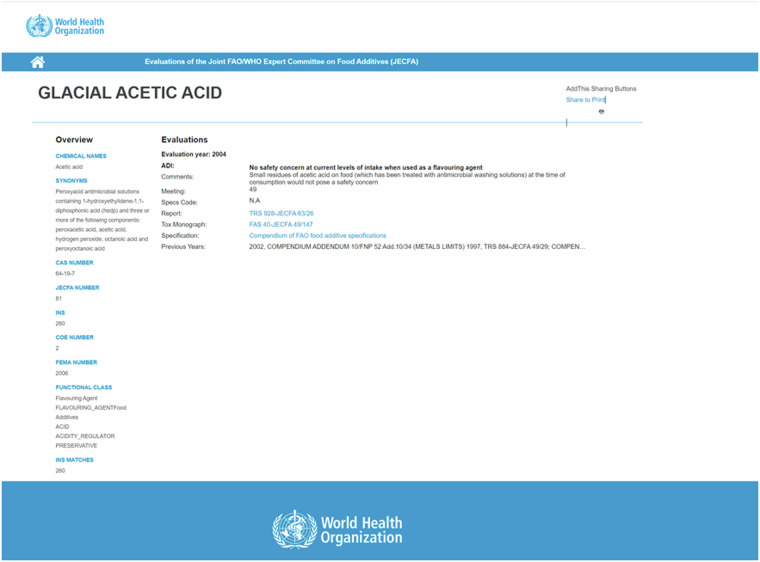
A sample page accessed on 03.02.2024 from https://apps.who.int/food-additives-contaminants-jecfa-database/Home/Chemical/1461. In this case, the JECFA name is ‘GLACIAL ACETIC ACID’, the Report is ‘TRS 928’ and the Report_sourcelink is https://iris.who.int/bitstream/handle/10665/43141/WHO_TRS_928.pdf;jsessionid=0DCA7842CC679C77E27CBC49E655FFA0?sequence=1.

We determined that iterating through potential URL endpoints (from 1 to 7000) would be the most efficient way to ensure comprehensive data coverage. This method was chosen after identifying that the JECFA database contains 6547 unique entries, which justified our approach of scanning a slightly broader range to capture all relevant data.The code constructs URLs, reads HTML content, and retrieves relevant information from web pages. In our scraping, we identified 6552 entries. We validated the effectiveness of this method by identifying 6552 entries, which were then cleaned to remove duplicates of (E)-N-[2-(1,3-BENZODIOXOL-5-YL)ETHYL]-3- (3,4-DIMETHOXYPHENYL)PROP-2-ENAMIDE (https://apps.who.int/food-additives-contaminants-jecfa-database/Home/Chemical/6316) and test pages, resulting in 6549 (https://apps.who.int/food-additives-contaminants-jecfa-database/Home/Chemical/5883) unique pages. This closely aligns with the expected number of entries, confirming the accuracy and efficiency of our approach. Figure 2 shows the fields of interest on a typical page accessible through the JECFA portal.

However, the content of the page varies, and we have identified in Table 1 a minimum set of information deemed relevant considering that it is common to most pages or necessary to establish a combination of identifiers. The code scrapes details related to chemicals and captures evaluation reports, links, and other data points. Using error handling ensures continued execution even if errors occur during the scraping process, providing additionally the printing of any encountered errors. Ultimately, it compiles the data collected into a dataframe and cleans it by removing empty variables and duplicates.

**Table 1:** Common content of most JECFA pages.

Contents	Description	Availability (%)
URL	A URL with the format https://apps.who.int/food-additives-contaminants-jecfa-database/Home/Chemical/n where the last ‘n’ is replaced by a number from 1 to 7000	100%
JECFA name	Name of the food additive within the JECFA database.	100%
Chemical names	Chemical names used to uniquely identify the substance	82%
Synonyms	Alternative names are used to refer to the same substance.	79%
CAS number	Unique numerical identifier assigned to each chemical substance in the Chemical Abstracts Service (CAS) Registry. It is structured in three parts separated by hyphens: a series of up to seven digits, a two-digit number indicating the total number of digits in the first part, and a single check digit in the third part.	35%
JECFA number	Identification number of the substance within the JECFA database.	71%
FEMA number	Identification number of flavoring substances evaluated and deemed safe for use in food by the Flavor and Extract Manufacturers Association (FEMA).	65%
COE number	Identification number assigned to food additives by the Codex Alimentarius Commission.	46%
Functional class	Categorizes food additives and contaminants based on their functional properties or roles in food processing (flavoring agents, food additives, food contaminants, and veterinary drugs).	98%
Evaluation year	Year in which the Joint FAO/WHO Expert Committee on Food Additives (JECFA) conducted a specific evaluation or assessment for a particular food additive or contaminant.	98%
Report	Provides users with access to comprehensive documentation of the evaluation process and findings for each substance^20^.	97%
Report sourcelink	Hyperlink that leads directly to the Report webpage	97%
Tox monograph	Provides users with access to comprehensive information on the toxicological evaluation of the substance^21^.	93%
Tox Monograph sourcelink	Hyperlink that leads directly to the Tox Monograph webpage	93%
Specification	Provides users with access to detailed information regarding the quality standards and requirements established for the substance.	92%
Specification sourcelink	Hyperlink that leads directly to the specification webpage	92%
FAS	It is a series of publications by the Joint FAO/WHO Expert Committee on Food Additives (JECFA). These series of documents contain toxicological evaluations of food additives, contaminants, natural occurring toxicants, and residues of veterinary drugs in food.	93%

## Data Records

The dataset is available at Figshare^16^ (10.6084/m9.figshare.26877211). As of May 2024, the final dataframe consists of 6552 rows.

The RDS file contains a structured data frame to reflect the fields described in Table 1 of the manuscript, including chemical names, identifiers, functional classes, evaluation years, and links to the corresponding reports and monographs. Each dataset entry corresponds to a unique chemical assessed by JECFA, providing a comprehensive resource for further analysis. This dataset is fully documented and can be explored using RStudio. For detailed instructions on how to use this dataset, please refer to the README file provided in the GitHub repository. This dataset enables targeted search and retrieval approach, enabling researchers to quickly locate the necessary documents for further data extraction and analysis. The dataset does not summarize the toxicological data itself. Instead, it provides an organized framework that researchers can use to locate specific documents within the JECFA repository, where they can perform more in-depth queries and extract the necessary toxicological details manually. For example, the EFSA inventory of BMR values for BMD analysis involved retrieving benchmark response (BMR) values from various international databases, including JECFA^12^. Using our automated indexing tool, researchers efficiently located relevant JECFA reports, which were then manually analyzed to compile the necessary BMR data.

The dataset retains the original content extracted from the JECFA database to ensure data integrity. Column headers have been standardized for better readability, while the data itself remain unchanged. Users are encouraged to further process the dataset according to their specific research needs.

Table 2 provides an overview of the evaluations conducted over different time periods and the classification of substances by their functional class. For the evaluation year, from 1958 to 2000 there were 2,356 evaluations, representing 37% of the total. Between 2000 and 2010, the number of evaluations increased significantly to 3,099 (48% of the total). The higher number of evaluations in previous years can be attributed to the initial establishment and rapid expansion of regulatory frameworks for food safety. When these systems were first implemented, there was a significant backlog of substances that needed to be evaluated to ensure that they met safety standards. In terms of functional class, the majority of the evaluated substances were flavoring agents, with 4,635 substances comprising 72% of the total. The predominance of flavoring agents among the substances evaluated can be explained by their widespread use in the food industry. The unknowns have only the JECFA name without associated compound names or additional information.

**Table 2:** Descriptive of the JECFA database.

Variable	Level	Statistics n (%)
Evaluation year	[1958–2000)	2,357 (37%)
	[2000–2010)	3,099 (48%)
	[2010–2020)	778 (12%)
	[2020–2023]	200 (3.1%)
	Unknown	118
Functional Class	Flavoring Agent	4,635 (72%)
	Food Additives	1,453 (23%)
	Food Contaminant	77 (1.2%)
	Veterinary Drug	277 (4.3%)
	Unknown	110

Although almost all 85 FAS codes are referenced, the ones collectively accounting for 50% of the records are 40, 42, 44, 46, 48, 50, and 52. We access and store all FAS toxicological monographs available on the Joint FAO/WHO Expert Committee on Food Additives (JECFA) website (https://www.who.int/groups/joint-fao-who-expert-committee-on-food-additives-(jecfa)/publications/toxicological-monographs) in PDF format, whenever available, or HTML as the 1st, 4th, 5th, 6th, 8th, 10th, and 12th through 52nd series of FAS monographs are available in HTML format only. WHO monographs beginning with the 51st series are also available in PDF format.

In cases where a FAS document is unavailable, the WHO TRS can be obtained by manually downloading it from the “Report_sourcelink” column in the pre-processed file.

## Technical Validation

To ensure the integrity of our data collection process, a validation exercise was carried out to assess whether the content of the website is consistent with the web scraping for all the variables listed in Table 1, thus confirming the reliability of the data set. A random sample of identifiers of 1 to 7000 was used to compose the URL, mimicking the process used to generate the URL accessed through web scraping. Given the absence of specific guidelines regarding the optimal proportion of samples for manual inspection, we tested 700 web pages (10%). This choice aligns with the limited existing literature on data validation and sampling methodologies in the context of web scraping validation^12,17^. Manual access to the web page was given to extract data and store them in an electronic data capture system, namely the Research Electronic Data Capture tool (REDCap®) hosted at the Unit of Biostatics and Public Health (University of Padova). Each variable on the web-scraped page was meticulously compared with its counterpart derived from manual inspection. The website was classified as a ‘failure’ if a discrepancy was found in at least one variable. The percentage of failures quantifies the error in the process. In particular, we observed minimal discrepancies, notably in only four contents across web pages that display the following percentages. Report (0.6%), Report sourcelink (0.9%), Tox Monograph sourcelink (5.3%), Specification sourcelink (0.2%)^18^.

Furthermore, an additional check was conducted on the JECFA database website (https://apps.who.int/food-additives-contaminants-jecfa-database/Home) using its search functionality by first character.

The search functionality of the portal for chemicals by the first character yielded 6,547 items. This number aligns with the number of websites containing at least a JECFA name (n = 6,551 minus a duplicate and a test page), suggesting that the process had not only a good positive predictive value but was also sensitive to the retrieval of the data.

## Usage Notes

This sequence of actions would enable the efficient retrieval and storage of PDF documents from the provided dataset, streamlining the process of data utilization and management:

- Data cleaning. The code would initiate its process by cleaning the ‘Report’ column within the data set. It would specifically target and remove any unwanted characters that appear at the beginning of the string entries. For example, if an entry starts with the characters “>”, the code would remove these first two characters. This step is crucial for resolving any formatting issues present in the data, ensuring that subsequent analysis is based on clean and accurate information.
- File name sanitization. The code would then define a function responsible for sanitizing file names. This function would replace any characters that are not allowed in file names with underscores. Additionally, it would trim any leading or trailing underscores to maintain a neat and standardized file naming convention. This is important to prevent errors when saving files and facilitate better organization of data resources.
- PDF URL extraction. Moving forward, the code would extract a list of PDF URLs from a specific column in the dataset, such as ‘Report_sourcelink’. These URLs would be collected into a vector and assigned to a variable, commonly referred to as PDF_urls. This collection of URLs serves as a gateway to accessing the documents needed for further analysis or review.
- PDF download. The final step would involve the code entering a loop to process each URL stored in the PDF_urls vector. It would perform several checks to ensure the validity and presence of each URL. If a URL is missing or improperly formatted, the code would skip it. For URLs that pass the checks, the code would construct a corresponding file name for the PDF and proceed to download the document using a web request function. Upon successful download (indicated by a status code 200), the code would save the PDF in a designated folder, such as “TRS”, using the constructed file name. The code would also handle scenarios where the download fails or the file already exists by logging appropriate messages.

The ability to clean data, sanitize file names, extract URLs, and download PDFs in batches is instrumental in unlocking the full potential of the dataset. It facilitates the creation of a streamlined workflow for data retrieval and post-processing, which can significantly enhance productivity and insight generation.

## Data Availability

The source code of the whole project is freely available on GitHub (https://github.com/UBESP-DCTV/jecfa), provided under MIT license. The project was developed in the R programming language (v4.3.2), powered by the ‘{targets}’ r-package^19^ (v1.4.1). The complete project, along with all required system and software dependencies, including the web-based Posit RStudio Server open IDE (Integrated Development Environment), is also available and executable as a self-contained Docker image on Docker-Hub (https://hub.docker.com/repository/docker/corradolanera/jecfa). The Dockerfile, docker-compose, and Makefile scripts used to create/update the image and run its container are available on the same GitHub repository mentioned. We have also developed a dedicated website that consolidates all relevant documentation and project resources. This website provides easy access to all materials and can be found at https://ubesp-dctv.github.io/jecfa/.
